# Time to change focus? Transitioning from higher neonatal to higher stillbirth mortality in São Paulo State, Brazil

**DOI:** 10.1371/journal.pone.0190060

**Published:** 2017-12-22

**Authors:** Kathryn Andrews, Maria Lúcia Moraes Bourroul, Günther Fink, Sandra Grisi, Ana Paula Scoleze Ferrer, Edna Maria de Albuquerque Diniz, Alexandra Brentani

**Affiliations:** 1 Department of Global Health and Population, Harvard T.H. Chan School of Public Health, Boston, Massachusetts, United States of America; 2 Children’s Institute of the Clinics Hospital, School of Medicine at the University of São Paulo, São Paulo, Brazil; 3 Swiss Tropical and Public Health Institute and University of Basel, Basel, Switzerland; 4 Department of Pediatrics, School of Medicine at the University of São Paulo, São Paulo, Brazil; TNO, NETHERLANDS

## Abstract

**Background:**

Differential trends in mortality suggest that stillbirths may dominate neonatal mortality in the medium to long run. Brazil has made major efforts to improve data collection on health indicators at granular geographic levels, and provides an ideal environment to test this hypothesis. Our goals were to examine levels and trends in stillbirths and neonatal deaths and the extent to which the mortality burden caused by stillbirths dominates neonatal mortality at the municipality- and state-level.

**Methods:**

We used data from the Brazilian Ministry of Health’s repository on births, fetal, and neonatal deaths (2010–2014) to calculate stillbirth and neonatal mortality rates for São Paulo state’s 645 municipalities.

**Results:**

At the state level, 7.9 per 1000 pregnancies ended in stillbirth (fetal death >22 weeks gestation or fetal weight >500g), but this varied from 0.0 to 28.4 per 1000 across municipalities. 7.9 per 1000 live births also died within the first 28 days. 42% of municipalities had a higher stillbirth rate than neonatal mortality rate, and in 61% of areas with low neonatal mortality (<8.0 per 1000), stillbirth rates exceeded neonatal mortality rates.

**Conclusions:**

This analysis suggests large variability and inequality in mortality outcomes at the sub-national level. The results also imply that stillbirth mortality may exceed neonatal mortality in Brazil and similar settings in the next few decades, which suggests a need for a shift in policy. This work further underscores the importance of continued research into causes and prevention of stillbirth.

## Introduction

While impressive improvements have been seen during the Millennium Development Goal (MDG) era in maternal and child health, improvements in stillbirths have been more limited; there were still an estimated 2.6 million stillbirths worldwide in 2015 [1], which is almost the same as the estimated number of neonatal death in the same year [2]. In fact, in contrast to child mortality, stillbirth reduction was never explicitly prioritized as part of the MDGs. To address this high burden, Goal Two of the 2014 *Every Newborn*: *An Action Plan to End Preventable Deaths* aims to reduce national-level stillbirth rates to 12 per 1000 births by 2030, and then to ten per 1000 births by 2035 [3]. *The Global Strategy for Women’s*, *Children’s and Adolescents’ Health (2016–2030)* further supports this by calling for an end to preventable stillbirths [4].

Despite calls to improve data collection and reporting [3, 5], reliable and consistent data on stillbirths are scarce [6–8]. This is partially driven by challenges in defining and identifying stillbirths consistently worldwide, and may contribute to the remarkable variation in stillbirth rates around the world [6]. While Finland had the lowest rate in 2015 at 1.1 per 1000 births and Pakistan had the highest at 43.1 per 1000 births, Brazil is cited as a success story in stillbirth reduction [1]. Specifically, Brazil’s stillbirth rate declined from 12.1 per 1000 births in 2000 to 8.6 per 1000 births in 2015 [1]. However, given the large degree of inequality within and across states in Brazil [9], these national-level statistics may mask substantial heterogeneity. While some studies have reviewed the literature on stillbirths in Brazil [10, 11] or presented results at the region- or state-level [12, 13], as of yet, no analyses of stillbirths in Brazil have been conducted at more granular geographic levels.

As neonatal mortality declines as a country becomes more developed, stillbirth rates are hypothesized to eventually equal and then exceed those of neonatal mortality [14]. This hypothesis exists because in general, reductions in neonatal mortality appear easier to achieve than reductions in stillbirth rates, meaning that a higher stillbirth burden seems likely in the long run [14]. However, as stillbirth reduction has not, until recently, been part of international goals, it is possible that this hypothesis is at least partially driven by a lack of effort or evidence. That said, as an example of these differential trends, a study of changes in stillbirths and neonatal deaths in Europe found that neonatal mortality declined by 29% between 2004 and 2010 while stillbirth rates only declined by 17% [15]. Further research is required to better understand the transition from a higher burden of neonatal deaths to a higher burden of stillbirths, which may help in prioritization and resource allocation for pregnancy, delivery, and post-delivery interventions.

Over the past ten years, the Brazilian Ministry of Health has made major efforts to improve the measurement of stillbirths. The Brazilian data available at low administrative levels allow for detailed sub-national analyses on stillbirth and neonatal mortality rates, and stratification of stillbirths by various fetal characteristics.

This paper is the first to systematically analyze these newly-available data on stillbirths. The goal of this analysis is threefold. First, we aim to examine the extent to which national- or state-level trends mask sub-regional variation in the incidence of stillbirths within the highly heterogeneous setting of São Paulo. Second, we aim to assess the general relationship between stillbirths and neonatal mortality (and the extent to which stillbirth mortality dominates neonatal mortality already), and third, we aim to establish the rates at which the mortality burden caused by stillbirths begins to dominate neonatal mortality on average.

## Methods

### Setting

Within Brazil, São Paulo state is the most populous, with a population of 44.4 million [16], and with its 645 municipalities [17], is a place of vast disparities, ranging from some of the most expensive neighborhoods of São Paulo municipality to some of the worst slums. S1 Fig shows where São Paulo municipality (the largest, with a population of 11.6 million [16]) is located within São Paulo state and where both are located within Brazil [18]. Our analysis focuses on São Paulo state and its municipalities.

### Data

For this analysis, we used data from the Brazilian Ministry of Health’s data repository [19] on deaths [20] and live births [21]. Vital statistics data are collected by the State Health Secretariats and reported to the Ministry of Health, where they are subject to quality improvement efforts, and are made publicly available organized by municipality of residence. Births are tracked by birth certificates, which are recorded in health facilities and in local registry offices (for births occurring outside of a health facility). Similarly, death certificates are completed in health facilities or morgues, or must be signed by a medical doctor and submitted to a notary’s office for deaths occurring at home (by law, no burial can take place without the official death certificate). The Program for Improvement of Death Cause Information in São Paulo (PRO-AIM) has managed the death registries of all municipalities in São Paulo since 1989, and has contributed significantly to data quality improvement. There is still some potential for underreporting of births and deaths (such as those outside of a health facility in a remote area, among individuals with extreme social exclusion, or due to illegally-induced abortions in clandestine clinics), which the Ministry of Health attempts to reduce by active search and by authorizing the completion of the death certificate by two witnesses in certain cases. However, 98% of births in Brazil take place in a facility [22], which suggests that underreporting of births and stillbirths is rare. While data are available for as early as 1996, major quality improvements in the data collection were implemented in 2010 [23–25]; we therefore restrict our analysis to use data from 2010 to 2014 (the most recent year available) on live births [26], infant deaths [27], and fetal deaths [28].

### Outcome measures

A stillbirth is defined by the International Classification of Diseases, 10^th^ revision (ICD-10) as a fetal death after 22 weeks of gestation, or a fetal death with a weight of over 500 grams [1]. We use this definition for our primary analysis, but also present stillbirth rates using the stillbirth definition used by the World Health Organization (fetal death after 28 weeks of gestation) [29] and another commonly used definition (fetal death with a birthweight of 1000 grams or more) [1] in S1 Text. Records of fetal deaths where both the gestational age and birthweight are missing cannot be classified as stillbirths, so are omitted from the primary analysis (we present alternative results including these unclassifiable fetal deaths in S1 Text).

We calculated stillbirth rates and neonatal mortality rates for each of São Paulo state’s 645 municipalities and for the state as a whole. Stillbirth rates were calculated as the number of stillbirths per 1000 births (live births plus stillbirths [30, 31]). Neonatal mortality rates were calculated as the number of deaths occurring in the first 27 completed days of life per 1000 live births, as is standard [32]. To reduce measurement error, we also present some results that are aggregated across 2010 to 2014, or that only include municipalities with at least 1000 live births. To provide a view into potential areas of focus to reduce stillbirths in São Paulo, we also extracted fetal death counts stratified by gestational age, weight, and timing of death (before or during delivery) from the same data repository.

### Statistical analysis

We calculate the number of municipalities where the stillbirth rate is statistically significantly different from the neonatal mortality rate, assuming both follow a Poisson distribution. In order to examine the approximate neonatal mortality rate at which stillbirth rates become higher than neonatal mortality rates, we use a locally-weighted linear regression (lowess) with default bandwidth of 0.8 to help visualize the relationship.

Stata/SE version 13.1 was used for data analysis, QGIS was used for map creation, and the municipal boundaries for the maps were from the Instituto Brazileiro de Geografia e Estatística [33]. Role of the funding source: Not applicable.

## Results

**Fig 1:**
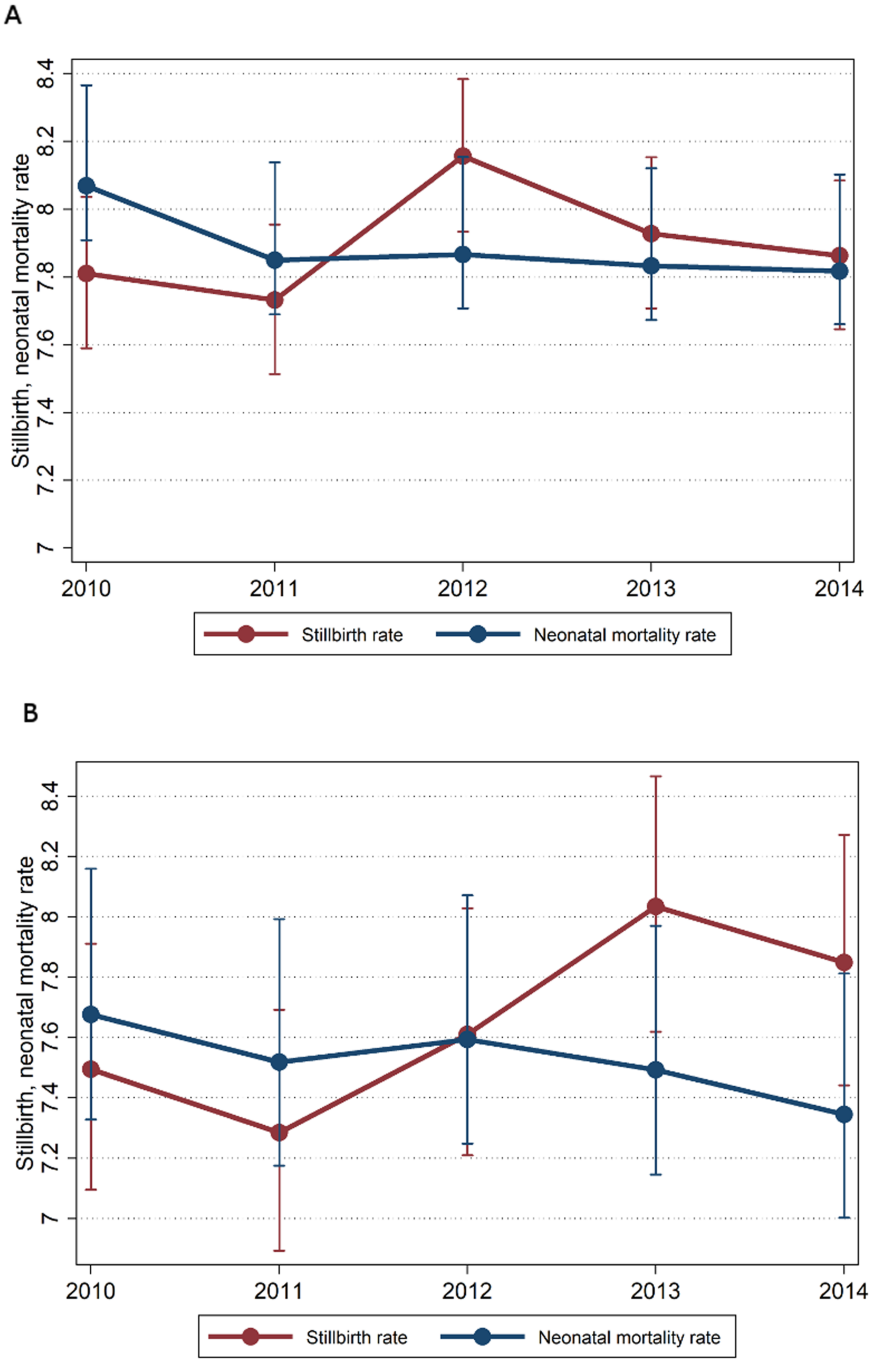
Trends in stillbirth and neonatal mortality rates (per 1000 births, with 95% confidence intervals) from 2010 to 2014 within (A) the entirety of São Paulo (SP) state and (B) São Paulo municipality only.

**Fig 2:**
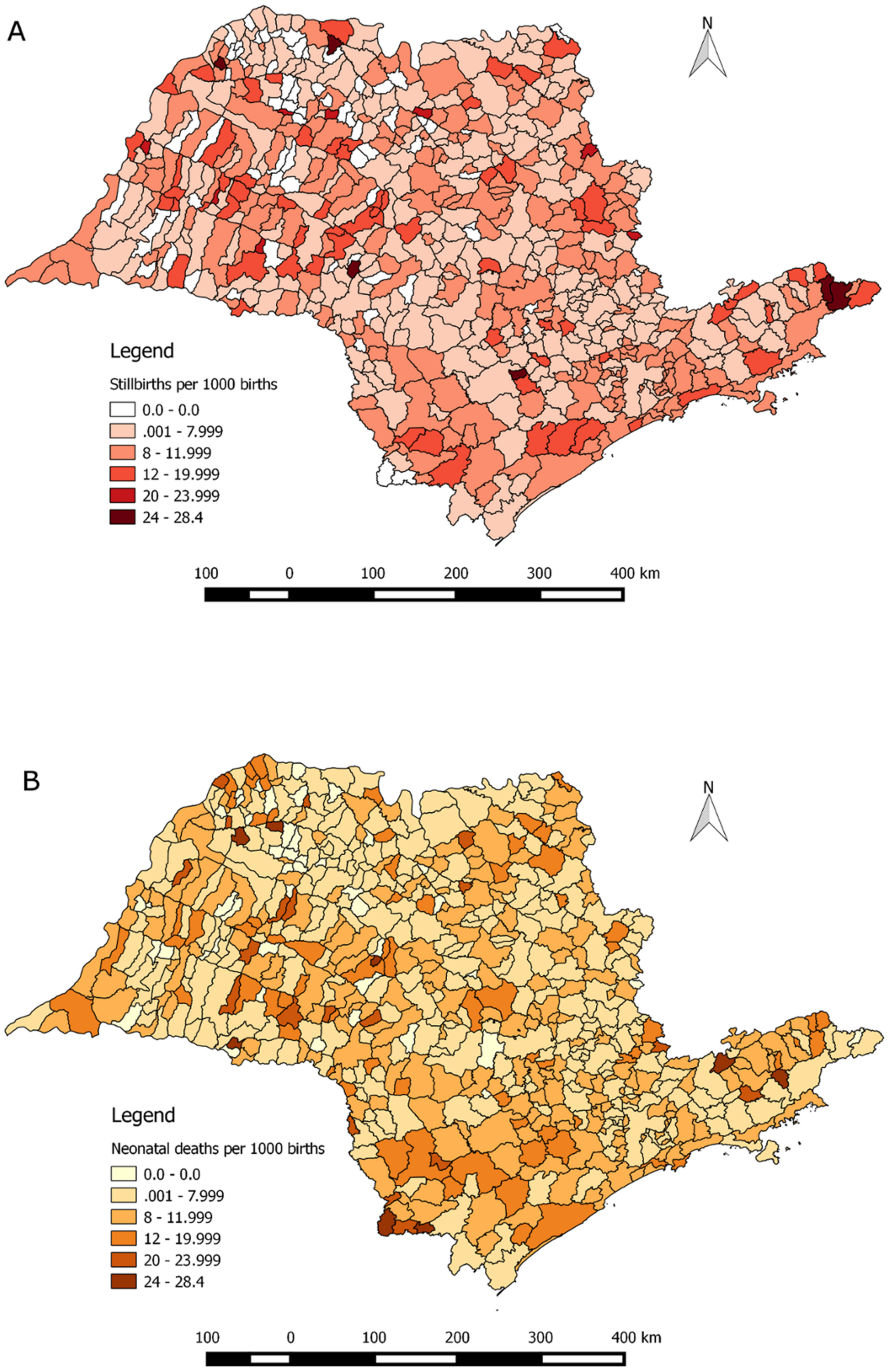
Stillbirths per 1000 births (A) and neonatal deaths per 1000 live births (B) aggregated across the years 2010–2014 in each of São Paulo’s 645 municipalities.

The total number of stillbirths in São Paulo state remained relatively stable from 4734 in 2010, to 4755 in 2011, 5071 in 2012, 4882 in 2013, and 4959 in 2014. The trend for São Paulo municipality was similarly flat, with 1316 stillbirths in 2010, 1295 in 2011, 1349 in 2012, 1401 in 2013, and 1391 in 2014. The stillbirth rate, aggregated across all 5 years, was 7.90 (95% confidence intervals [CIs] 7.80, 8.00) per 1000 births in São Paulo state and only marginally lower at 7.65 (95% CIs 7.47, 7.84) per 1000 births in São Paulo municipality. Fig 1 shows stillbirth rates over time for the state (panel A) and municipality (panel B) of São Paulo, contrasted against neonatal mortality rates, and suggests a small uptick in stillbirth rates in São Paulo municipality since 2011. At the municipality level, stillbirth rates ranged from 0 to 28.44 stillbirths per 1000 births, with a mean of 7.60 and a median of 7.56 per 1000 births. There were 69 (of 645) municipalities that had no reported stillbirths over the five-year period of this analysis. It is important to note that the 8 municipalities with the highest stillbirth rates had small sample sizes (fewer than 10 stillbirths over the analysis period). Panel A of Fig 2 shows the municipality-specific stillbirth rates. The two darkest shades on Panel A’s map indicate the 81 municipalities where the stillbirth rate is higher than the upcoming global target for year 2030 of 12 per 1000 births. There remain 161 municipalities that fail to meet the subsequent 2035 target of fewer than ten stillbirths per 1000 births.

Overall, the neonatal mortality rate was slightly higher than the stillbirth rate, with a mean 8.21 and a median of 7.84 per 1000 live births across all 645 municipalities (though with a similar range of 0 to 28.13 per 1000 live births). There were 45 municipalities that had no reported neonatal deaths over this analysis period. São Paulo municipality in particular had a neonatal mortality rate of 7.58 (95% CIs 7.40, 7.77) per 1000 live births when aggregating across years 2010 to 2014, and São Paulo state had a rate of 7.95 (95% CIs 7.85, 8.05) per 1000 live births during the same period. Panel B of Fig 2 shows the municipality-specific neonatal mortality rates.

**Fig 3:**
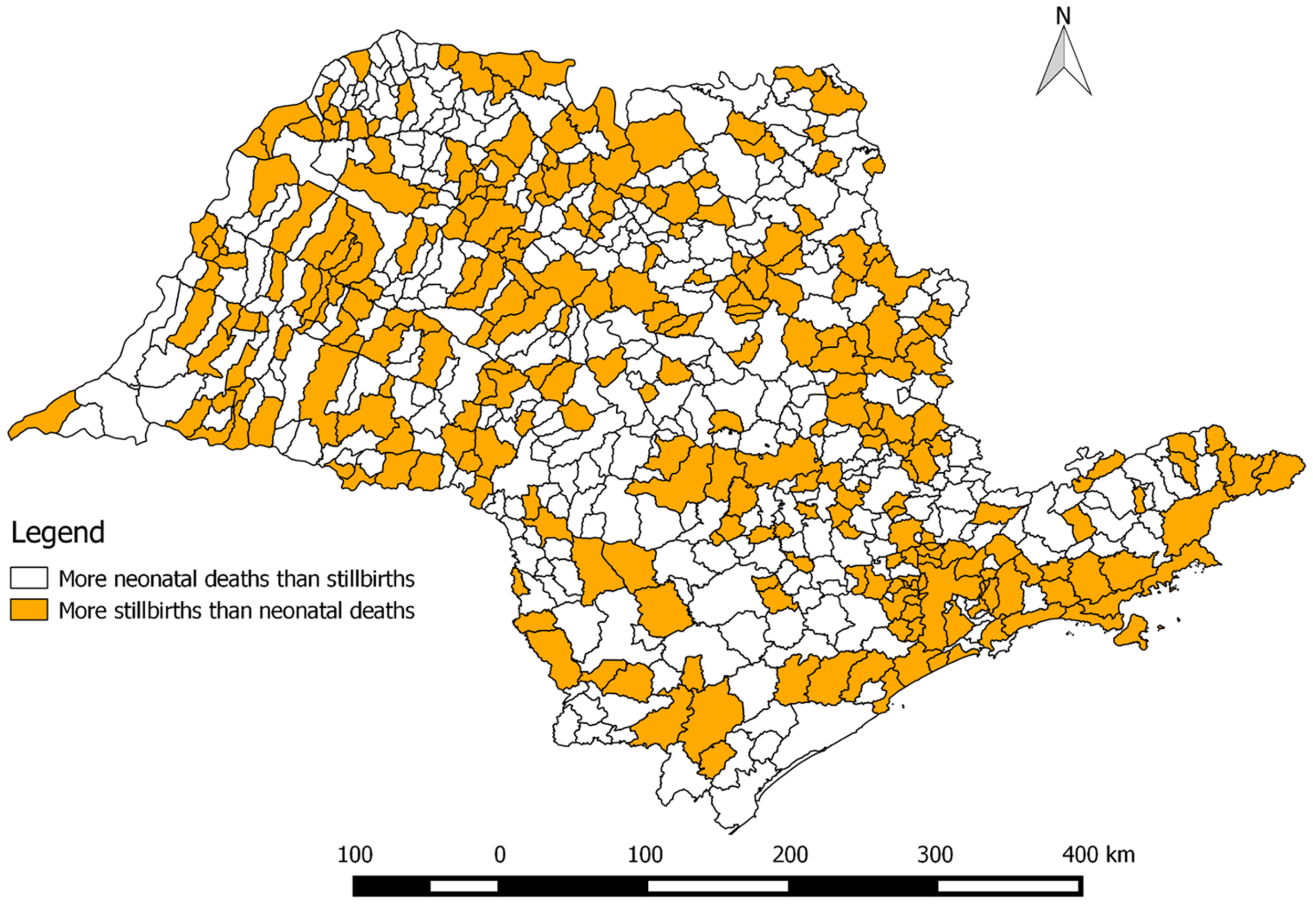
273 (of 645) municipalities in the state of São Paulo where there were more stillbirths than neonatal deaths over the period 2010–2014.

**Fig 4:**
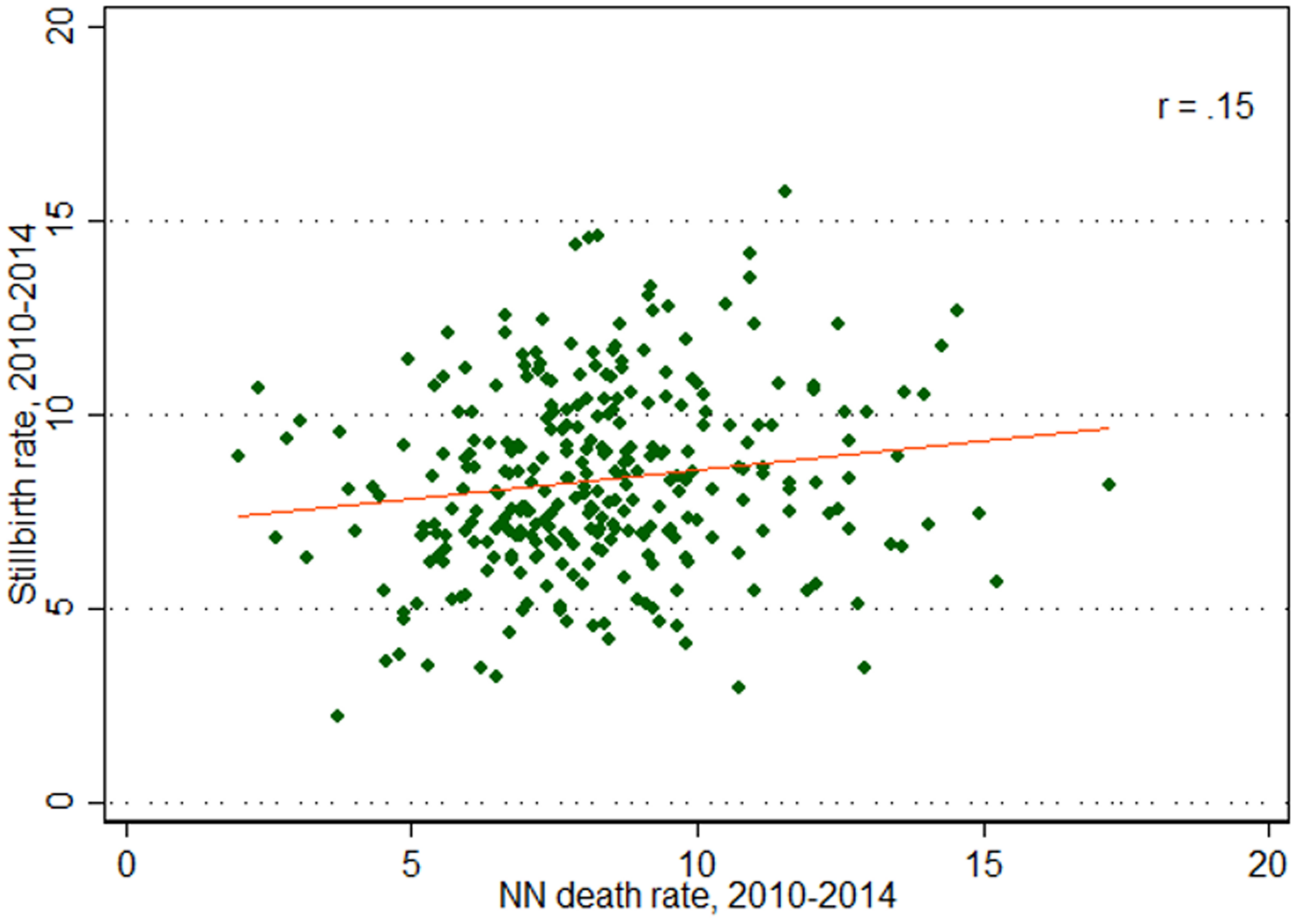
Comparison of stillbirth rate and neonatal death rate over 2010–2014 for 296 municipalities with >1000 live births, with a linear regression line to show the slope of the relationship.

Despite the comparable state-level stillbirth and neonatal mortality rates, in 273 municipalities (of 645, or 42%), there were more stillbirths than neonatal deaths over the years 2010 to 2014 (highlighted in Fig 3). However, in only 94 municipalities was the difference between the stillbirth and neonatal mortality rate statistically significant (not accounting for multiple testing). The mean difference was nearly zero (0.03 more neonatal deaths than stillbirths), but the values ranged from 177 more neonatal deaths than stillbirths in São Paulo municipality to 114 more stillbirths than neonatal deaths in Sorocaba municipality. Fig 4 compares rates of stillbirths to rates of neonatal deaths (aggregated from 2010 to 2014) across the municipalities of São Paulo state just for those municipalities with greater than 1000 live births during this period, to reduce the measurement error associated with small sample sizes. This relationship between neonatal mortality and stillbirth rates is rather weak but positive (correlation coefficient of 0.15, p-value < 0.0001).

**Fig 5:**
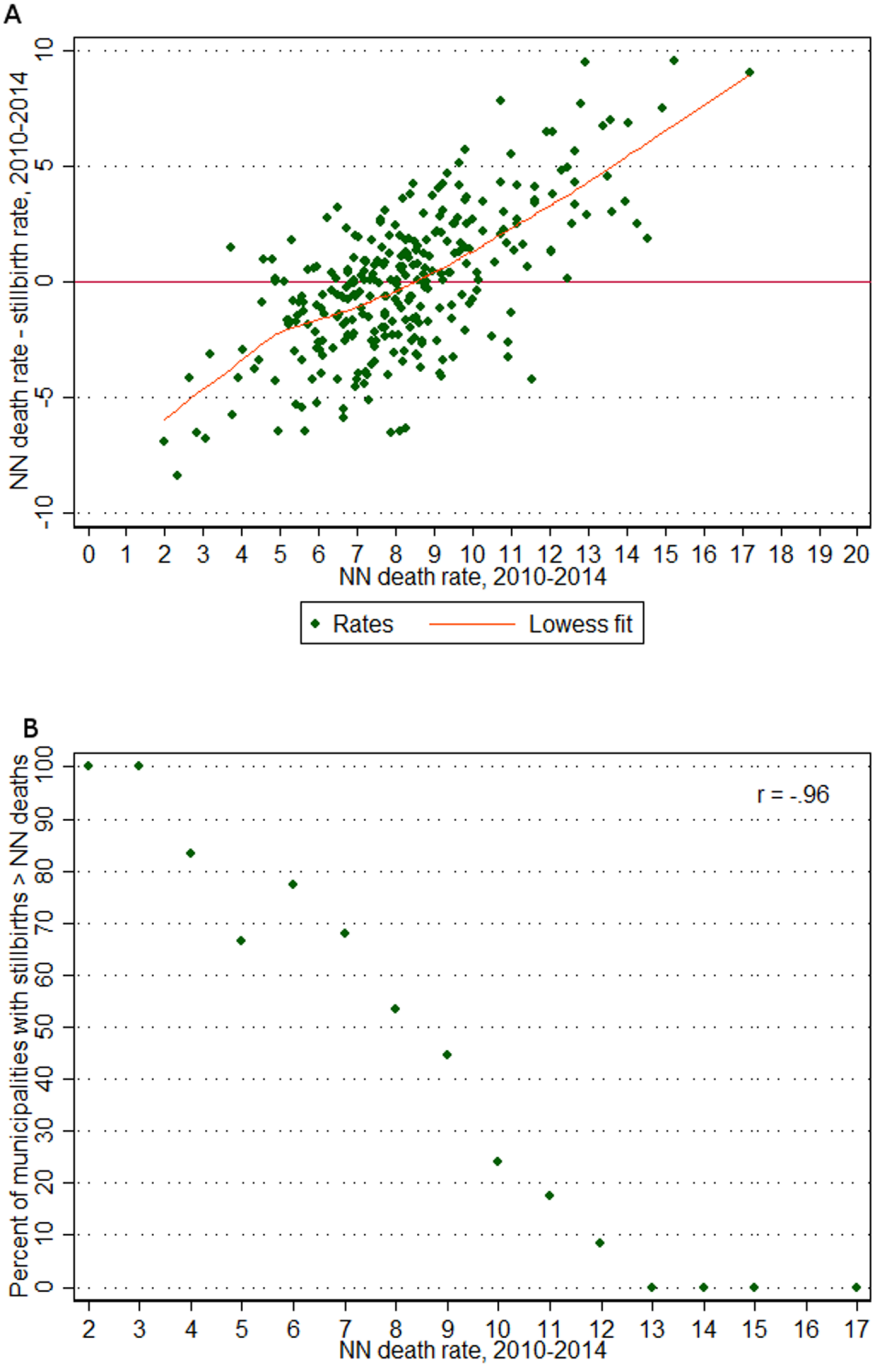
Among 296 municipalities with >1000 live births over 2010–2014, (A) difference between neonatal (NN) death rate and stillbirth rate compared to neonatal death rate, and (B) percent of municipalities with more stillbirths than neonatal deaths by neonatal death rate (rounded to nearest whole number).

Panel A of Fig 5 shows the results of subtracting the stillbirth rate from the neonatal mortality rate (across 2010 to 2014) in each municipality with greater than 1000 live births and comparing it to the neonatal mortality rate. This strong, positive relationship indicates that at high rates of neonatal mortality, the neonatal mortality rates are higher than stillbirth rates, but at low rates of neonatal mortality, stillbirth rates are more likely to dominate. Specifically, the lowess model fit indicates that between eight and nine neonatal deaths per 1000 live births (where the lowess line intersects the horizontal line indicating zero difference between neonatal death and stillbirth rates) is the approximate average point below which stillbirth rates become higher than neonatal mortality rates. When neonatal mortality is below nine per 1000 live births, 57% of municipalities have higher stillbirth rates than neonatal mortality rates, and this rises to 61% among those areas with neonatal mortality below eight per 1000.

At present, 51% of municipalities have neonatal mortality rates below eight per 1000 live births (when aggregating across 2010 to 2014). If all municipalities were to follow the national-level 25-year trend in annual rate of reduction in neonatal mortality of 3.85% (based on a decline from 24 to nine neonatal deaths per 1000 live births between 1990 and 2015 [32]), we would expect 84% of municipalities to have neonatal mortality rates of fewer than eight per 1000 live births after ten years, and 96% after 20 years. If the relationship between neonatal mortality and stillbirths observed here holds, this would suggest that increasing fractions of municipalities would have higher stillbirth rates than neonatal mortality rates after ten and 20 years, respectively.

Panel B of Fig 5 shows the decline in the percent of municipalities with a higher stillbirth rate than neonatal mortality rate as neonatal death rates increase (correlation coefficient of -0.96, p-value < 0.0001). It is clear that among municipalities with low neonatal death rates, stillbirth rates dominate (with 100% of municipalities with neonatal mortality rates of three per 1000 or less having higher stillbirth rates than neonatal mortality rates). Where neonatal death rates are higher, neonatal mortality dominates (with all municipalities with neonatal death rates of 13 per 1000 or greater having higher neonatal death rates than stillbirth rates).

In order to better understand potential areas of focus to reduce stillbirths, we present characteristics of fetuses in Table 1. Across 2010 to 2014, the percent of stillbirths with normal birthweight (≥2500g) ranges between 15% and 20%; a similar percent of stillbirths are full term (>36 weeks gestation). The percent of fetal deaths that occur during birth remains at 3% or lower across all years. As visible by the counts in the “Unknown” categories, an important fraction of fetal deaths are missing key information on characteristics.

**Table 1:** Characteristics of fetal deaths by year, aggregated across São Paulo state.

		Year				
		2010	2011	2012	2013	2014
Weight at birth	Under 500g	176 (3%)	258 (5%)	362 (6%)	380 (7%)	439 (8%)
	500 to 999g	1473 (28%)	1428 (27%)	1517 (27%)	1421 (26%)	1498 (27%)
	1000 to 1499g	741 (14%)	809 (15%)	812 (14%)	828 (15%)	854 (15%)
	1500 to 2499g	1112 (21%)	1147 (22%)	1295 (23%)	1240 (22%)	1160 (21%)
	2500 to 2999g	490 (9%)	431 (8%)	461 (8%)	490 (9%)	479 (9%)
	3000 to 3999g	463 (9%)	496 (9%)	460 (8%)	447 (8%)	464 (8%)
	4000g and above	85 (2%)	94 (2%)	75 (1%)	98 (2%)	76 (1%)
	Unknown	639 (12%)	576 (11%)	729 (13%)	615 (11%)	617 (11%)
Gestational age	Less than 22 weeks	287 (6%)	359 (7%)	527 (9%)	507 (9%)	539 (10%)
	22 to 27 weeks	1166 (23%)	1104 (21%)	1108 (19%)	1033 (19%)	1175 (21%)
	28 to 31 weeks	899 (17%)	911 (17%)	989 (17%)	940 (17%)	923 (17%)
	32 to 36 weeks	1444 (28%)	1304 (25%)	1330 (23%)	1389 (25%)	1389 (25%)
	37 to 41 weeks	873 (17%)	947 (18%)	952 (17%)	909 (16%)	910 (16%)
	42 weeks and above	19 (0%)	14 (0%)	19 (0%)	12 (0%)	16 (0%)
	Unknown	491 (9%)	600 (11%)	786 (14%)	729 (13%)	635 (11%)
Timing of stillbirth	Before birth	3781 (73%)	4678 (89%)	4850 (85%)	4778 (87%)	4856 (87%)
	During birth	178 (3%)	0 (0%)	168 (3%)	127 (2%)	106 (2%)
	Unknown	1220 (24%)	561 (11%)	693 (12%)	614 (11%)	625 (11%)

## Discussion

While Brazil appears to be on-track at the country-level to achieve stillbirth rate targets, the results presented in this paper suggest a remarkable amount of mortality disparities at the municipality level. The disparities between the municipalities with the lowest rates and those with the highest are similar to the disparities between the highest income countries and the poorest countries of sub-Saharan Africa. Specifically, municipalities range from zero stillbirths over the course of the five years examined to stillbirth rates equivalent to Ethiopia’s (29.7 per 1000 births [1]). *The Lancet*’s 2016 *Ending Preventable Stillbirths Series* may cite Brazil as a success story in that it is no longer on the top ten list for number of stillbirths worldwide as of 2015, but the results presented here highlight the importance of not allowing national-level achievements to overshadow inequalities at the local level. It remains important to ensure that impressive gains do not produce complacency with respect to addressing sub-national areas that require additional support. In order to further improve the national-level stillbirth rate statistics, it may become increasingly important to address pockets of high stillbirth rates such as those identified here. Further examination of the risk factors and determinants of stillbirths in high-risk municipalities will be required to reduce disparities.

This analysis identified that areas of high stillbirth rates generally coincide with areas of high neonatal mortality rates, suggesting that certain municipalities may require additional support to reduce both fetal and early infant death. This may be in the form of efforts to reduce maternal risk factors/behaviors and/or improve health system quality to address the antepartum and intrapartum needs of women. Perhaps more interestingly, the finding that areas of high neonatal mortality rates generally have higher neonatal death rates than stillbirth rates, in addition to the finding that areas of lower neonatal death rates are more likely to have larger stillbirth rates than neonatal death rates, is indicative of the transition described previously. Specifically, we find that 61% of municipalities with neonatal mortality rates less than eight per 1000 live births have already transitioned to higher stillbirth than neonatal mortality rates, and we project that almost 60% of all municipalities will have higher stillbirth than neonatal mortality rates in 20 years. If we speculate that the findings from the highly heterogeneous environment of São Paulo state could be applicable elsewhere, this suggests that stillbirth mortality may soon dominate neonatal mortality in middle- and high-income countries. While some interventions can prevent both stillbirths and neonatal deaths (such as appropriate obstetric care, fetal monitoring for asphyxia, and prevention/treatment of maternal infections [34, 35]), still others mainly address neonatal mortality only (such as ventilators, artificial surfactant, and Kangaroo Mother Care [34–36]). Under conditions of limited resources, these findings suggest that it may be appropriate to transition policy attention in the areas that have already made the transition to higher stillbirth rates toward interventions that target more than just neonatal deaths.

The small fraction of stillbirths that occur during labor in São Paulo state (ranging from zero to four percent of fetal deaths across 2010 to 2014 where timing of demise is known) mirror trends in developed countries as a whole, where ten percent of stillbirths are estimated to occur intrapartum [1]. Intrapartum stillbirths are typically attributable to lack of high-quality delivery care [1, 37] (of note, over 98% of deliveries occur in a facility in Brazil [22]), while antepartum stillbirths are more closely tied to maternal risk factors such as hypertension, obesity, diabetes, infection, and smoking, in addition to placental abnormalities and fetal growth restriction [38–40]. The pattern seen in São Paulo state further emphasizes the findings from previous work in Brazil [39] suggesting that efforts to reduce stillbirths going forward will need to focus on these gestational risks. Since over 90% of pregnant women in Brazil attend at least four antenatal care visits [41], this suggests the presence of opportunities for risk reduction through clinic-based intervention.

In 2013, among fetal deaths with known weight or gestational age, 21% of stillbirths in São Paulo state were among fetuses with a normal birthweight (more than 2500g) and 19% had a gestational age of at least full term (more than 36 weeks of gestation). These values are comparable to the same statistics in the United States in the same year (18.9% and 19.2%, respectively) [42], and suggest that substantial reductions in stillbirth rates could be achieved by focusing on this subset. This finding further underscores the importance of continued research into the causes of late/full term stillbirths, many of which remain “unexplained” [40].

Given the difficulty of collecting accurate data on stillbirths, this analysis is limited by the available data. The most recent year of available data is 2014, which does not allow us to examine more recent trends, such as any changes in the last years since the global stillbirth targets were released in 2014. The definition of stillbirths used here requires that we know either the gestational age or the birthweight of the fetus in order to classify the fetal death as a stillbirth. This means that 1,614 fetal deaths between 2010 and 2014 with missing gestational age and birthweight in the Ministry of Health database were unclassifiable. An unknown fraction of these were stillbirths and had they been included in this analysis, may have identified additional municipalities with high stillbirth rates (in S1 Text we present a sensitivity analysis assuming all unclassifiable deaths were stillbirths). While 1,614 unclassifiable fetal deaths may seem small in contrast to the total 24,401 classifiable stillbirths in this analysis, there is ample room for improvement in the quality of data collection. Relatedly, over the study period, there were a total of 31 stillbirths, 12 neonatal deaths, and 61 live births in São Paulo state where the municipality was unknown. While we included these in the aggregate state-level estimates of stillbirth and neonatal mortality rates, additional areas with high rates may have been identifiable if the municipalities of these deaths had been known. This analysis is also limited to the births and deaths that are reported to the Ministry of Health; as described previously, there is potential for some underreporting in particularly remote or marginalized areas. In addition, given the small administrative level examined in this analysis, we chose to aggregate stillbirth counts across four years to provide more stable results. This necessarily limits us, however, in examining municipality-specific trends over time, and masks any recent improvements in stillbirth rates. As mentioned previously, some of the municipalities with the highest stillbirth rates are subject to small sample sizes, which is why we present some results using only municipalities with more than 1000 births over the study period to increase the stability of the results. Finally, this analysis focuses on the largest of Brazil’s 27 states, but the findings are not generalizable to the other 26.

Despite its limitations, this analysis provides a unique and important contribution to the current stillbirths literature. It is the first to examine stillbirths in the specific context of São Paulo, to use the new detailed data available to highlight sub-national disparities in stillbirth rates, and to empirically examine the transition from higher neonatal death rates to higher stillbirth rates (further, finding a specific average point when the transition occurs). Further examination of the risk factors and determinants of stillbirths in high-risk municipalities will be required to reduce the disparities seen here. The massive efforts on behalf of the Brazilian Ministry of Health to make these high-quality in-depth data available are commendable and are a positive step toward answering recent calls to push forth the research agenda to help bring an end to preventable stillbirths.

## Supporting information

S1 Fig São Paulo municipality highlighted within São Paulo state highlighted within Brazil [18]. (TIF)

S1 Text Description and visualization of stillbirth rates using alternative definitions of stillbirth. (DOCX)
