# Supplementary figures and images for: Time to change focus? Transitioning from higher neonatal to higher stillbirth mortality in São Paulo State, Brazil
Source: PLoS One. 2017 Dec 22;12(12):e0190060. doi: 10.1371/journal.pone.0190060 (PMC5741246; doi:10.1371/journal.pone.0190060)

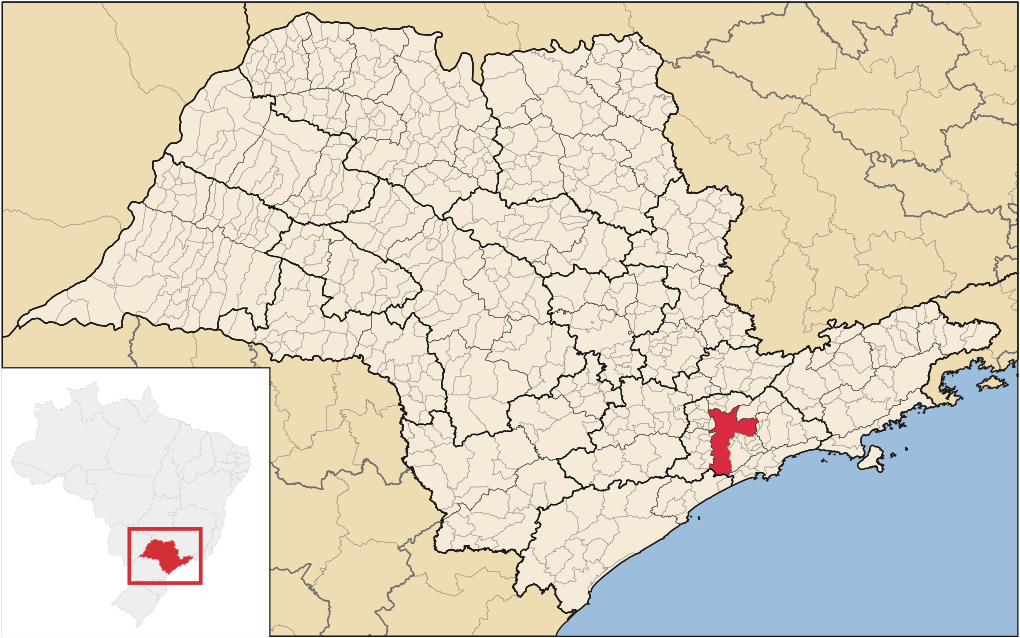

Supplement: S1 Fig — (TIF) [file pone.0190060.s001.tif]
