# Supplementary material for: Time to change focus? Transitioning from higher neonatal to higher stillbirth mortality in São Paulo State, Brazil
Source: PLoS One. 2017 Dec 22;12(12):e0190060. doi: 10.1371/journal.pone.0190060 (PMC5741246; doi:10.1371/journal.pone.0190060)
Supplement: S1 Text — (DOCX) [file pone.0190060.s002.docx]

**S1 Text for** *Time to change focus? Transitioning from higher neonatal to higher stillbirth mortality in São Paulo State, Brazil*

Due to the frequency with which various definitions of stillbirths are used, we present here stillbirth rates and counts based on two other commonly-used definitions. Figures 1 and 2 can be compared to main text Figure 2 (Panel A) to view how the different definitions affect municipality-level stillbirth rates. The definition used in the main analysis (based on ICD-10) results in slightly more stillbirths than the two alternatives discussed below in that it includes some fetal deaths that the alternative definitions consider to be miscarriages rather than stillbirths. Therefore, our more inclusive definition as used in the main analysis yields a total of 24,401 stillbirths in the state of São Paulo between 2010 and 2014, which is greater than the number of stillbirths using the two definitions described below.

The first alternative definition is based on the World Health Organization’s definition of stillbirth being a fetal death after at least 28 weeks of gestation [1]. Using this definition, there were a total of 16,189 stillbirths during 2010-2014 in the state of São Paulo, which corresponds to a rate of 5.25 stillbirths per 1000 births. The stillbirth rate across the 645 municipalities ranged from 0 to 24.10, with a mean of 5.48 and a median of 5.26 stillbirths per 1000 births. There were 28 municipalities with stillbirth rates greater than the international goal of 12 per 1000 births. This municipality-specific rates are visible in Figure 1 below.

The second alternative definition of stillbirth is a fetal death with a birthweight of 1000 grams or more [2]. Using this definition, there were a total of 15,107 stillbirths during 2010-2014 in the state, corresponding to a stillbirth rate of 4.91 stillbirths per 1000 births. The stillbirth rate across the 645 municipalities ranged from 0 to 24.10, with a mean of 5.28 and a median of 4.90 stillbirths per 1000 births. Based on this definition, there were 31 municipalities with stillbirth rates greater than the goal of 12. This municipality-specific rates are visible in Figure 2 below.

We also demonstrate the impact of including the “unclassifiable” fetal deaths in our estimates of stillbirth rates. In order to be classified as a stillbirth, the birthweight or gestational age of a fetus must be known. If both pieces of information are missing, the fetal death could have been either a miscarriage or a stillbirth. There were 1,614 fetal deaths between 2010 and 2014 where both birthweight and gestational age were missing; an unknown fraction of those deaths were likely stillbirths. As a sensitivity analysis, in Appendix Figure 4, we present stillbirth rates assuming all unclassifiable fetal deaths were in fact stillbirths. This resulted in a total of 25,954 stillbirths during 2010-2014 in the state, corresponding to a stillbirth rate of 8.42 stillbirths per 1000 births. The stillbirth rate across the 645 municipalities ranged from 0 to 29.41, with a mean of 8.11 and a median of 8.10 stillbirths per 1000 births. Based on this definition, there were 100 municipalities with stillbirth rates greater than the goal of 12. This municipality-specific rates are visible in Figure 3 below.

**Figure 1**: Stillbirths (defined based on WHO’s definition of fetal deaths at 28 weeks or greater gestation) per 1000 births aggregated across the years 2010-2014 in each of São Paulo’s 645 municipalities


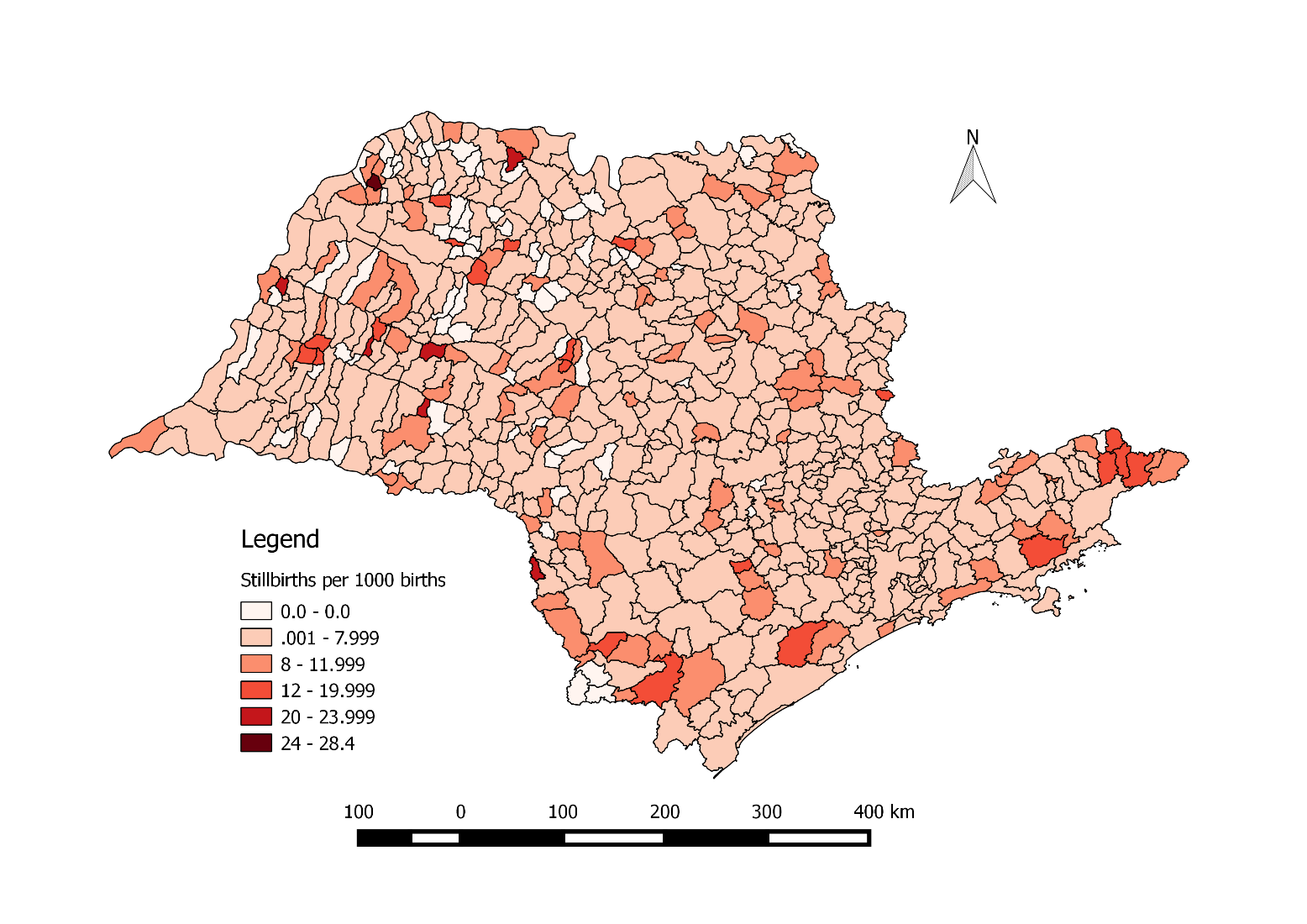


**Figure 2**: Stillbirths (defined as fetal deaths with birthweight of at least 1000 grams) per 1000 births aggregated across the years 2010-2014 in each of São Paulo’s 645 municipalities


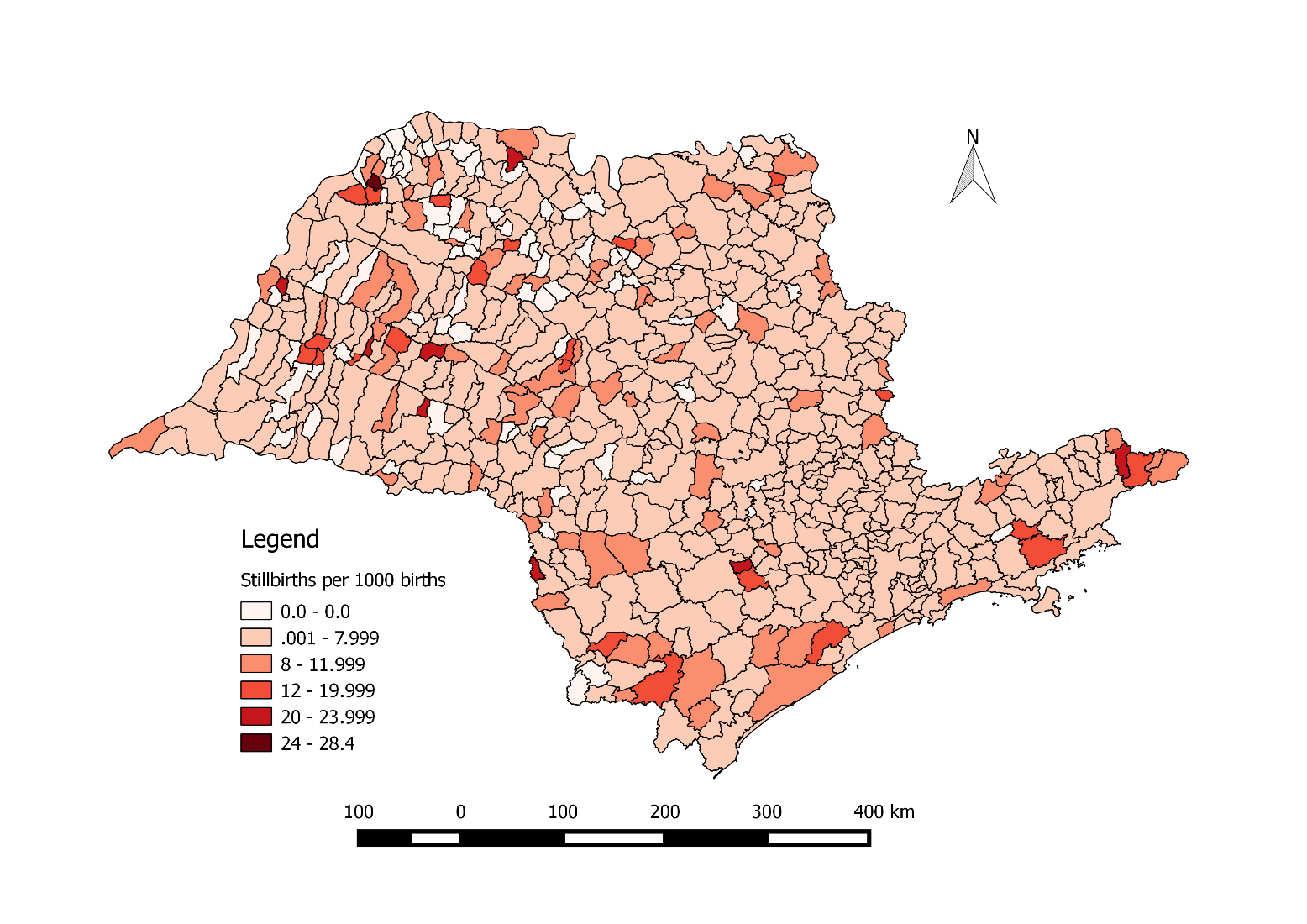


**Figure 3**: Stillbirths (defined as fetal deaths after 22 weeks of gestation or a with a weight of over 500 grams, or a fetal death when both gestational age and weight are missing) per 1000 births aggregated across the years 2010-2014 in each of São Paulo’s 645 municipalities


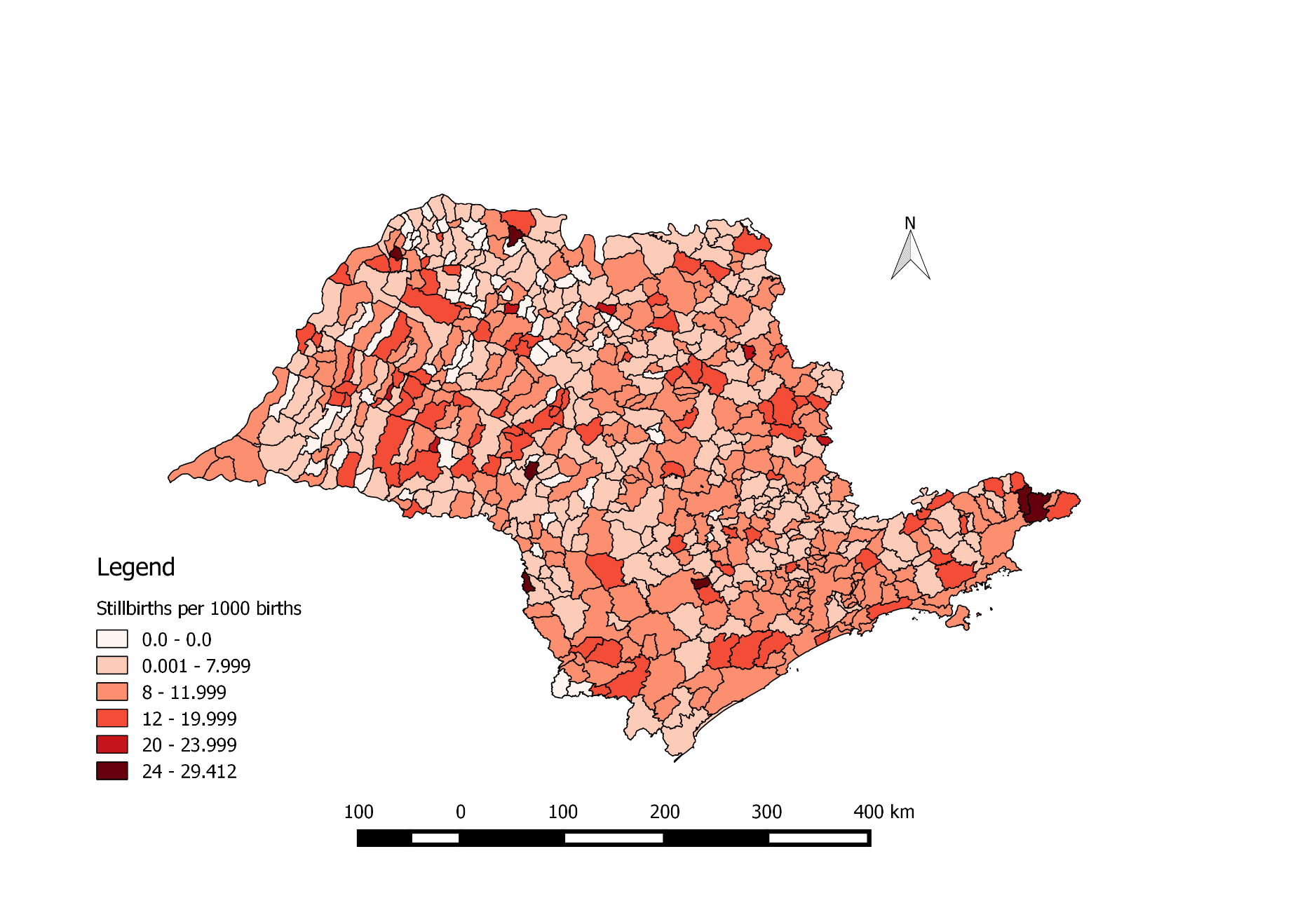


**References**

1. World Health Organization. WHO | Stillbirths. WHO. 2016. http://www.who.int/maternal_child_adolescent/epidemiology/stillbirth/en/. Accessed 2 May 2016.

2. Lawn JE, Blencowe H, Waiswa P, Amouzou A, Mathers C, Hogan D, et al. Stillbirths: rates, risk factors, and acceleration towards 2030. The Lancet. 2016;387:587–603.
